# Cystic Degeneration of the Chorion, with Uterine Hydatids

**Published:** 1878-02

**Authors:** L. Humphreys

**Affiliations:** South Bend, Indiana


### CYSTIC DEGENERATION OF THE CHORION, WITH UTERINE HYDATIDS.

BY L. HUMPHREYS, M. D., SOUTH BEND, INDIANA.

At 4 a. m. Oct. 24th, 1877, I was called to see Mrs. M., aged 29, who was supposed to be in premature labor, at about the sixth month of her utero-gestation.

The patient was having irregular uterine pains and very free hemorrhage, saturating cloths under and about her person, and a quantity of coagula was found near the vulva.

Removing the coagula, an examination per vaginam revealed the os dilated to the size of a quarter of a dollar, and within it a spongy mass that appeared to the touch to be placental. The neck of the uterus was conical and about 1¼ inches in length. The hand upon the abdomen discovered the fundus considerably above the umbilicus, and the abdominal enlargement was about equal to that of the sixth month of gestation. No solid contents could be felt within the uterus through the abdominal walls; pressure revealed only a soft, yielding mass. I was unable to determine the nature of the case at this time, and proceeded at once to tampon the vagina; gave two fluid drachms of the extract of ergot, applied firm pressure over the fundus of the uterus, and awaited results. In 20 or 30 minutes the uterine contractions were increased, and the vaginal packing and a mass of hydatids, enveloped in a dark colored membranous sac, were expelled. The sac was about 3 inches in its short and 6 or 7 inches in its long diameter, was ruptured in passing the os and vaginal canal, and from it clusters of hydatids protruded. Their expulsion was accompanied and followed for a short time by alarming hemorrhage. Grasping the body of the uterus over the abdomen, the contractions of that organ could be distinctly felt, and in another half hour fully three pints of grape-like cysts were discharged, followed by a considerable quantity of coagula. The cysts were about the size of and bore a striking resemblance to pale green Malaga grapes. Some were amber colored and others white.

The hydatids were pediculated and attached by delicate membranous filaments, looking much like clusters of grapes. When the uterus had well contracted, indicating that its anomalous contents were expelled, the usual bandage was applied, and no unpleasant effects resulted. The lochial discharge, in character and quality, was that of natural parturition at full term. The secretion from the mammary glands was quite free for several days, requiring the use of artificial means to relieve them. The patient remained in bed ten or twelve days, and is now in excellent health. She possesses a remarkably fine physique, is 140 pounds in weight, symmetrical in body and limbs, temperament lymphatic.

Some authorities are of the opinion that uterine hydatids occur most often in women of a lymphatic temperament.

This patient had been married six years. About two years ago she aborted at between the second and third months, and was troubled with frequent uterine hemorrhages for some weeks after leaving her bed. These ceased, only after the removal of the remains of a retained or adherent placenta, by her attending physician. When this last supposed utero-gestation began, she passed her second period without any appearance of menses. At the third month there was a slight uterine flow, and soon after this profuse hemorrhage set in, and continued at intervals of a week or ten days, with the frequent extrusion of large quantities of coagula. From the commencement of gestation, Mrs. M—— suffered from persistent nausea and vomiting, almost day and night, rejecting almost all food and medicine. For some time nourishment per rectum was the only means by which strength and vitality could be maintained.

From loss of blood and want of proper nourishment, the patient became decidedly anæmic and much reduced in strength. About two months prior to the time of my first seeing her, she visited friends in this city, leaving her home in Northwestern Michigan, and traveling by rail the entire distance. Soon after her arrival here, the stomach regained its functions, and her appetite became excellent, her strength and general health rapidly improved; in fact, she was quite restored to ordinary health, except as regarded the periodic uterine hemorrhages. This restoration of health and strength was most fortunate for the patient before the expulsion of the uterine hydatids, else in all probability she would have sunk, from exhaustion and loss of blood, if she had continued in her former anæmic condition. I may add, that in a practice of nearly forty years, with a fair share of obstetric business, the case here reported, is only the second of its kind occurring in my own observation. I saw one other in consultation, in the practice of another physician.

---

At a meeting held January 24th, the Medical Board of the Illinois Charitable Eye and Ear Infirmary, nominated Prof. Edmund Andrews as consulting surgeon, to fill the vacancy created by the resignation of Prof. E. Powell.

